# Inferring the Spatio-temporal Patterns of Dengue Transmission from Surveillance Data in Guangzhou, China

**DOI:** 10.1371/journal.pntd.0004633

**Published:** 2016-04-22

**Authors:** Guanghu Zhu, Jiming Liu, Qi Tan, Benyun Shi

**Affiliations:** 1 Department of Computer Science, Hong Kong Baptist University, Kowloon Tong, Hong Kong; 2 School of Mathematics and Computing Science, Guilin University of Electronic Technology, Guilin, China; 3 School of Computer Science and Technology, Hangzhou Dianzi University, Hangzhou, China; Santa Fe Institute, UNITED STATES

## Abstract

**Background:**

Dengue is a serious vector-borne disease, and incidence rates have significantly increased during the past few years, particularly in 2014 in Guangzhou. The current situation is more complicated, due to various factors such as climate warming, urbanization, population increase, and human mobility. The purpose of this study is to detect dengue transmission patterns and identify the disease dispersion dynamics in Guangzhou, China.

**Methodology:**

We conducted surveys in 12 districts of Guangzhou, and collected daily data of Breteau index (BI) and reported cases between September and November 2014 from the public health authority reports. Based on the available data and the Ross-Macdonald theory, we propose a dengue transmission model that systematically integrates entomologic, demographic, and environmental information. In this model, we use (1) BI data and geographic variables to evaluate the spatial heterogeneities of Aedes mosquitoes, (2) a radiation model to simulate the daily mobility of humans, and (3) a Markov chain Monte Carlo (MCMC) method to estimate the model parameters.

**Results/Conclusions:**

By implementing our proposed model, we can (1) estimate the incidence rates of dengue, and trace the infection time and locations, (2) assess risk factors and evaluate the infection threat in a city, and (3) evaluate the primary diffusion process in different districts. From the results, we can see that dengue infections exhibited a spatial and temporal variation during 2014 in Guangzhou. We find that urbanization, vector activities, and human behavior play significant roles in shaping the dengue outbreak and the patterns of its spread. This study offers useful information on dengue dynamics, which can help policy makers improve control and prevention measures.

## Author Summary

Dengue transmission is a spatio-temporal process with interactions between hosts, vectors, and viruses. Its transmission also involves multiple complex or even hidden factors, such as climate, social environment, vector ecology, and host mobility. These complexities make the underlying process of dengue transmission difficult to clarify. We address how the patterns of dengue transmission can be inferred by investigating the 2014 dengue outbreak in the city of Guangzhou, China, taking the available surveillance data and applying mathematical models and computational methods. We can then estimate the distribution of dengue infections and identify the transmission mechanisms. In our study, we systematically investigate the critical factors, enabling us to estimate the real patterns and dynamics of dengue transmission beyond the surveillance data.

## Introduction

Dengue is a mosquito-borne disease caused by one of the four dengue virus serotypes (DENV 1–4), and is primarily transmitted by Aedes aegypti and Aedes albopictus [1, 2]. The virus and its vectors are now widely distributed throughout tropical and subtropical regions, resulting in about half the world’s population being at risk of infection [1]. The World Health Organization (WHO) has estimated that 50–100 million infections occur annually in over 100 endemic countries [1, 3]. More recently, Bhatt et al. took into account the inapparent infections and found that the global burden is probably much higher, at about 390 million infections per year [4]. The problem of dengue epidemics in China has intensified over the past two decades [5], and between 1991 and 2013 about 21,532 dengue cases and 620 deaths were reported. In 2014, the incidence reached a peak, with 46,864 reported cases, 80% of which were infected in Guangzhou. Dengue is not endemic in China [5], but the current situation has become more complicated, and the exact causes of the increase in incidences and the detailed transmission characteristics are unclear [5].

In this study, we aim to identify the spatio-temporal transmission patterns of dengue epidemics in Guangzhou in 2014 by addressing the following questions: How can we estimate the temporal and geographical distributions of infection cases? How can we evaluate the infection risk and the control effects? How can we assess the interactions of the factors involved among different geographical locations and thus infer the diffusion process from one location to another? The answers to these questions will be influential in epidemiological inference and public health planning. Clear information about disease burden and infection risk can help in correctly evaluating the effects of the factors involved and the correct allocation of resources for intervention [4]. An accurate reflection of the transmission dynamics and diffusion process of dengue can help us more fully understand and further predict the prevalence of epidemic propagation. There are, however, many challenges to be addressed, such as misreported surveillance data, obscure vector indices, the hidden effects of hosts and vectors, and the heterogeneous infection processes. These challenges and existing works related to our questions are discussed in more detail below.

First, disease surveillance data is usually the baseline for estimating the infection burden, but it does not directly reflect the full extent of infection, for the following reasons: (1) multiple factors, such as inapparent infections, under-reporting, and misdiagnosis can lead to the misreporting of infected cases [4, 6]; (2) the incubation period of the dengue virus can create a delay between infection and reporting; (3) human mobility can lead to the mis-registration of infection locations [7]. Feasible techniques to handle these issues have been proposed, such as analyzing index clusters and serologic testing to evaluate inapparent dengue infections [8], using epidemiological models with exposed states (e.g., SEIR model) to account for the incubation period [9], and incorporating human mobility into the transmission model to examine its effect on dengue infection [10]. Epidemiological information can be extracted from hidden infections and unclear data through statistical and stochastic methods [11]. Ster et al. deployed reversible jump MCMC methods to reveal hidden infections and inferred the infectivity profile of the U.K. 2001 foot and mouth epidemic [12]. By fitting the partially observed data sequences of hospital infection, Cooper et al. estimated key epidemiological parameters using a structured hidden Markov model [13]. We take the distribution of reported cases as the essential data, and also incorporate the factors of incubation time lag, host mobility and reported rate, to estimate the 2014 actual dengue burden in Guangzhou.

Second, dengue infection risk is in reality primarily evaluated by vector indicators, such as the house index (HI), the container index (CI), the Breteau index (BI), the pupa index (PI), and the adult productivity index [2]. However, the traditional vector indices (e.g., HI, CI, and BI) have been shown to be poor proxies for measuring adult mosquito abundance and dengue risk, possibly due to the inadequate quality of the vector and incidence data, diversity of vector indices and adult vector densities, or to geographic/temporal mismatches of infection sites and index records [14–16]. Most vector indices only reflect vector prevalence rather than abundance [17], as they do not take into account the container type productivity. In view of this, other vector indicators have been suggested, such as sampling adult mosquitoes [14, 15, 18], or integrating vector indices and other information (e.g., combing demographics and indices [18]. Beyond the vector indices, standard notions have been proposed to assess infection potentials, such as the basic reproduction number [19, 20], the vectorial capacity (VCAP) [21–23], and the entomological incubation rate (EIR) [22, 24]. In this study, we systematically integrate environmental and ecological factors and BI data to evaluate the adult vector densities.

Third, the large-scale spread of dengue viruses is often caused by the spatial and temporal dynamics of vectors and hosts [25–27]. A combination of elements, including dense populations, frequent human-vector contact, rural-urban migration, serotype circulation, and inadequate infrastructure, can lead to dengue infection and mosquito breeding opportunities [16, 28, 29]. These factors are significant in shaping the spatial and temporal transmission of dengue epidemics. Two types of studies have been performed to reveal transmission process and assess the relevant factors [30, 31]. First, mapping techniques and statistical methods can be used to process various data, such as geographic information systems method [32], time-series Poisson regression [29], Moran’s *I* statistic [33, 34], and spatial scan statistics [35]. These methods can identify the hot spots, evaluate the relationship between different factors (e.g., climate, imported cases and urbanization) and dengue incidence, and estimate the dispersion process [29, 32–35]. The second type of analysis methods is primarily based on mathematical or computational models, and focus more on the intrinsic biting-based transmission process and the interaction between hosts, vectors, and viruses. These include differential equations [19, 20], spatially agent-based transmission models [36], and metapopulation models [10]. These methods are able to estimate the infection capacity and simulate the time evolution and spatial diffusion of dengue epidemics [10, 19, 20, 36]. However, it has been suggested that existing models less take into account the heterogeneity of mosquito densities/behaviors and mosquito-host encounters [31, 37], so may not effectively reflect the spatial heterogeneity and temporal variation in the transmission [25, 31].

**Fig 1:**
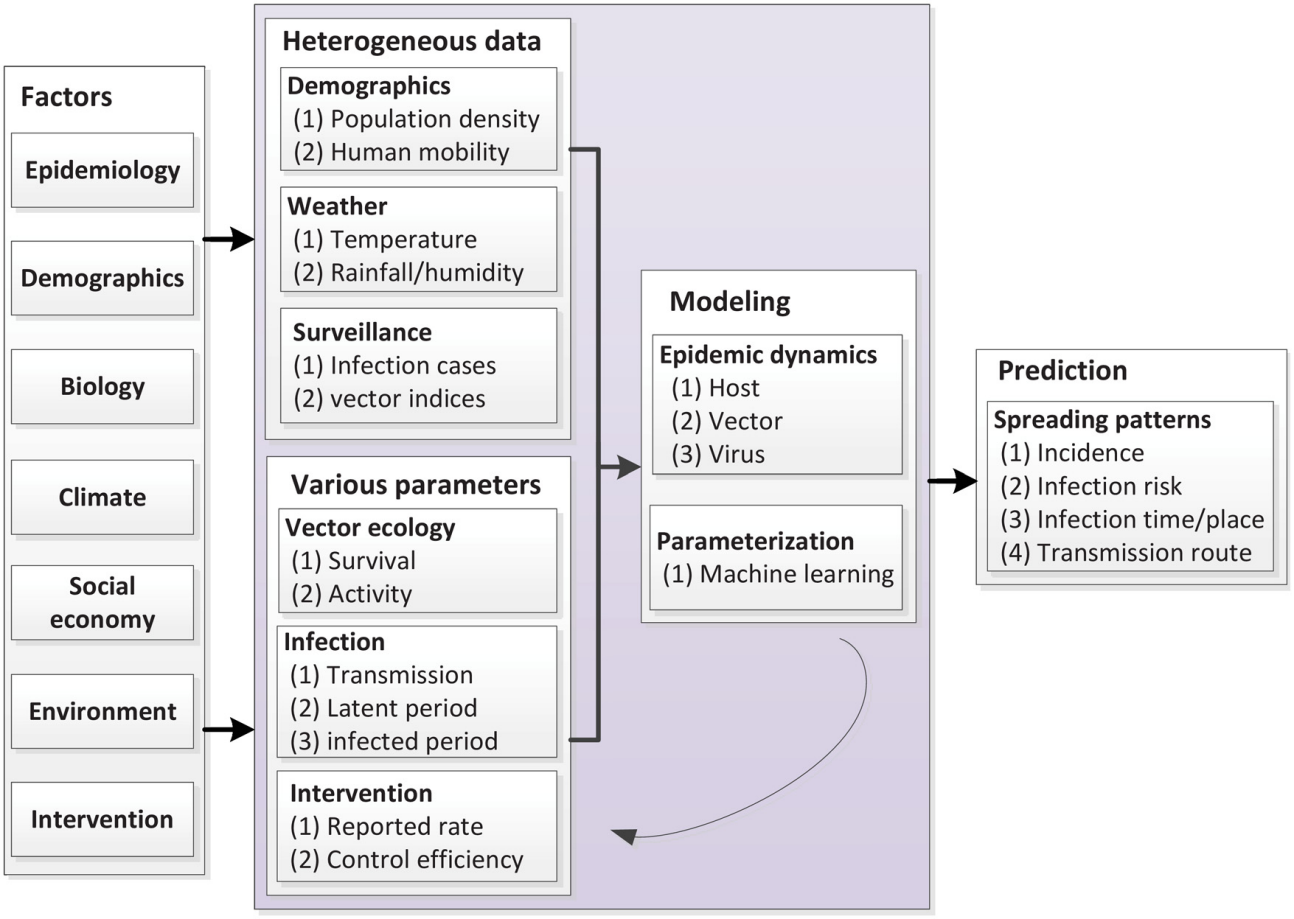
An illustration of the research framework. Taking multiple factors into consideration, we establish mathematical models (integrating data and parameters) that fit underlying parameters through computational methods, and then predict the spatio-temporal patterns of disease transmission.

In this study, we use mathematical models and computational methods to tackle the aforementioned problems. First, based on the Ross-Macdonald theory [9, 21–23], a transmission model is defined to simulate the spatial diffusion of the dengue virus. Second, large-scale and consecutive vector indices, together with environmental and ecological information, are integrated to estimate the vector quantities. Third, human mobility as a spatio-temporal driver of dengue spreading dynamics [36], is estimated based on the radiation model proposed by Simini et al. [38]. Underlying transmission parameters are quantified by fitting the model to real-world observations using machine learning methods, such as the Markov chain Monte Carlo (MCMC) method [39]. The issue of incomplete surveillance data is addressed by comparing the estimated incidence rates and surveillance data with a reported rate. The research framework is shown in Fig 1.

We conduct an empirical study in Guangzhou, where serious dengue epidemics have recently been experienced, particularly in 2014. Guangzhou is an international metropolis located in the tropical/subtropical region, and its climate and geography are ideal for vector growth and virus survival. In Guangzhou, the population is densely concentrated in the urban areas, with frequent movement between districts. By implementing the proposed model, we aim to (1) estimate the actual incidences and reveal the effects of environment and urbanization on vector activities; (2) evaluate the infection risk in terms of the basic reproduction number and explain the temporal pattern of infection potential in each district; (3) identify the underlying transmission process and mechanisms of dengue in Guangzhou.

## Materials and Methods

To modeling the spatio-temporal patterns of dengue transmission in Guangzhou, we first surveyed the situations about dengue outbreak (e.g., reported cases, control strategies and possible causes of outbreak) from the local government reports, newspaper, and other media, and then investigate the related information (e.g., geographical environment, vector indices, climate, population and transportation) in this region. Based on that, we propose a transmission model. Detailed procedure is presented in the following.

### Study areas

The city of Guangzhou (112°57′E to 114°3′E and 22°26′N to 23°56′N) is the capital of Guangdong province in South China, and has an area of 7434 square kilometers and about 12.93 million residents. The climate is humid and subtropical, with high temperatures and humidity in summer, and comparatively mild and dry in winter. The annual mean temperature is 22°*C* and the annual accumulate precipitation is 1,800 mm. Guangzhou is an international port and an important foreign trade gateway into China. The above information is based on the Guangzhou government site (http://www.gz.gov.cn/gzgov/s2289/zjgz.shtml).

**Fig 2:**
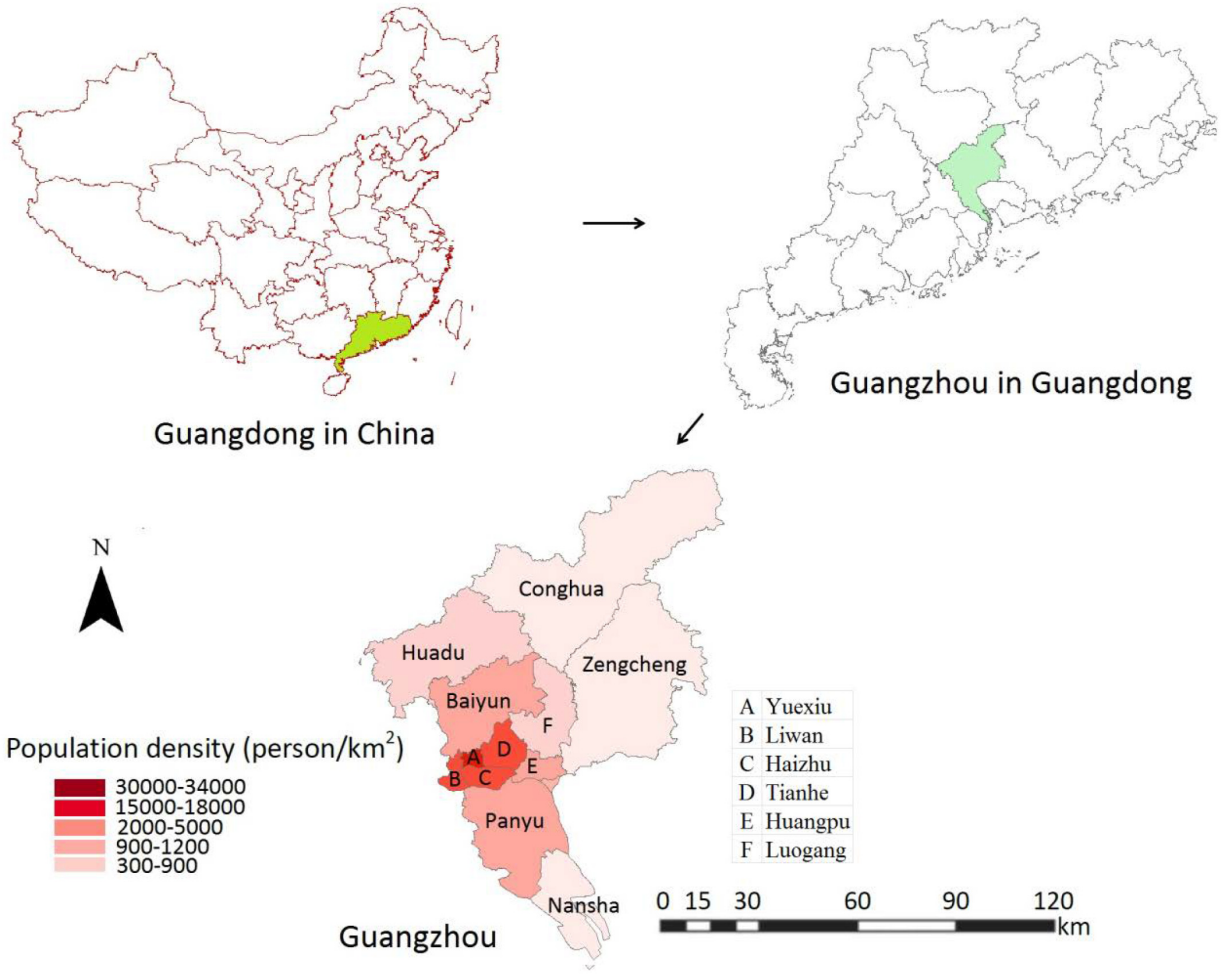
An illustration of the study areas. Guangzhou is the capital of Guangdong province in China and is composed of 12 districts.

Guangzhou consists of 12 districts and is divided into three areas: urban, suburban, and exurban, which follows the city’s overall planning (2010–2015) and its Five-Year Plan (2011–2015) that take into account the urbanization, population density, and green coverage. The urban areas are Liwan, Yuexiu, Haizhu, Tianhe, and Baiyun (south of Liuxihe and north second ring), which account for 3.8% of the city area, 46.6% of the total population, and about 56% of transportation. The suburban areas are Panyu, Huangpu, Luogang, Huadu, and Baiyun (outside the central area), and the exurban areas are Nansha, Zengcheng, and Conghua. A detailed map is shown in Fig 2.

### Data collection and parameter settings

To identify the underlying transmission patterns of dengue in 12 Guangzhou districts, the following data are collected.

- *Dengue cases.* Records of dengue daily cases in 12 districts are obtained from the Health Department of Guangdong Province (http://www.gdwst.gov.cn/). According to the “Law of the People’s Republic of China on the prevention and treatment of infectious diseases,” all dengue cases confirmed by any medical institution or hospital must be reported to the surveillance system in the local area within one day (12 hours in cities and towns, 24 hours in villages). However, some cases go unreported, and the records of infection time and place of some may be misreported [6].
- *Breteau index.* In Guangzhou, the available vector indicator is the BI data. Approximately 287 surveillance sites are distributed throughout the city, and the daily reports of BI in each district are collected by the Guangzhou Center for Disease Control and Prevention (http://www.gzcdc.org.cn/). In the affected areas, any indoor and outdoor water containers near any of 50 to 100 houses in the vicinity (outside the houses, a radius of 10m is considered as part of the property) are inspected. Larval growth is closely associated with temperature and rainfall [40], so the missing BI is estimated by stepwise linear regression using one week’s weather records.
- *Population density.* The population size in each district is retrieved from the 2013 Guangzhou Statistical Yearbook, but some of the population in certain districts (registered as residents) may travel to other districts early in the morning for work or study and return in the late afternoon.
- *Transportation.* The transportation data is taken from the Guangzhou Transport Development Annual Report and the Guangzhou traffic site (http://www.gzjt.gov.cn/gzjt/web/Default.aspx), from which the daily commuting level of the districts can be evaluated.

The parameters are set as follows.

- *Study time period.* Our study time period in Guangzhou is from September to November 2014. In January 2014 the imported cases have been found in Guangzhou, but the first autochthonous case was reported in June, and starting from August the local infections dominated the transmission.
- *Time step.* The time step is set to be one day, and each time step is divided into daytime and nighttime. The duration of daytime is set to be from 0700 (7 a.m.) to 1900 (7 p.m.), according to the working hour and commuting time in Guangzhou. It is assumed that residents who work in other districts stay there during the daytime, and that all residents stay in their home districts at night.
- *Biting rate during daytime and nighttime.* The dominant vector of dengue transmission in Guangzhou is Aedes albopictus. It has been reported that the Aedes mosquito is a diurnal feeder with peak biting periods in the early morning and in the evening [1], but surveys have shown that the biting activity of Aedes albopictus varies from place to place. For example, in Malaysia the activity peak is observed between 0600–0900 and 1500–2000 [41], and in Macau (near Guangzhou), between 0600–0800 and 1800–2000 [42], but in India, it shifts to 2230–2300 and 2030–2100 [43]. In our study, we assume that the biting rate during daytime and nighttime is the same, but has different values in different districts. The biting rate will be estimated by our modeling approach and machine learning methods.

### Transmission model

In this section, we propose a mathematical model integrating the geographic, transportation, demographic, environmental, and surveillance information in dengue transmission.

The relationship between incomplete surveillance data and the estimated number of incidences is discussed below. Surveillance systems usually record disease incidences in different locations as a set of time series, so if we have observed the incidences of *H* locations during time period *t* = 1, 2, ⋯, *T*, and the spatio-temporal surveillance data at time *t* are denoted by a vector Γ_*t*_ = (*γ*_1*t*_, *γ*_2*t*_, ⋯, *γ*_*Ht*_)^*T*^, then the correspondence of incidences between reporting and modeling can be quantified as follows:

$$\Gamma_{t}=\delta \rho_{t}+\varepsilon_{t},\;\varepsilon_{t}∼N\left(0,\Sigma \right),$$ (1)

where *δ* is the reported rate, *ρ*_*t*_ = (*ρ*_1*t*_, *ρ*_2*t*_, ⋯, *ρ*_*Ht*_)^*T*^ is the numbers of incidences derived from the modeling approach presented below, and *ε* is the error term, which follows normal distribution with variations $\Sigma =\text{diag}(\sigma_{1}^{2},\sigma_{2}^{2},\ldots ,\sigma_{H}^{2})$.

To specify and locate the infection events, the time step is set to be one day, and each is divided into daytime and nighttime, to take daily commuting and biting difference into account. For simplicity, the notations of subscript and superscript correspond to the district number and vector age, and the hat and check correspond to daytime and nighttime, respectively.

#### Vector density

To evaluate the infectious bites, we quantify the spatial densities of vectors (i.e., Aedes mosquitoes). The vector flight range is less than 400m, and biting behavior usually occurs around their habitats [1, 2], so we assume that the Aedes mosquitoes stay in their original locations. We further assume that the abundance of immature female vector is proportional to that day’s BI with parameter *K*, thus we can estimate the density of female Aedes mosquitoes with specific ages in each district. Let $x_{i}^{j}\left(t\right)$ denote the density of female adult vectors with age *j* at time *t* in district *i*. It is then calculated as

$$x_{i}^{j}\left(t\right)=KB_{i}\left(t-j-1\right)p\left(j\right),$$ (2)

for *j* = 1, 2, ⋯, *h*, where *B*_*i*_(*t*) is the value of BI at time *t* in district *i*. The other parameters are described in Table 1. The densities of adult female vectors $x_{i}\left(t\right)=\sum_{j}x_{i}^{j}\left(t\right)$ in district *i* is therefore dependent on the coefficient *K*, the BI values in early (1 + *h*) days, and the vector survival rates.

**Table 1:** Model parameters for the study in Guangzhou.

Parameters	Definition	Distribution	Source
*h*	The maximum of life span of adult vectors	44d	[44]
*p*(*i*)	Daily survival probability of adult mosquitoes at age *i*	Weilbull	[44]
*a*	Human blood feeding rate at 12 hours	Fitting^2^	
*k*	Extrinsic incubation period (EIP)	9d	[45]
*d*	The longest intrinsic incubation period (IIP)^1^	8d	[46]
*σ*	Age at which adult mosquitoes begin biting hosts	3d	[23]
*b* = *c*	Transmission probability from host to vector (=vector to host)	0.4	[47]
*δ*	The report rate^3^	0.25	[4]
*K*	The proportionality coefficient between BI and vector density	Fitting^2^	
*P*_*I*_	The probability distribution of infection period	Gamma(25, 0.2)	[19, 48]

^1^The IIP follows Log-normal distribution denoted as *P*_*L*_ [46].

^2^These parameters can be fitted by machine learning methods.

^3^The reported rate is adopted from the proportion of symptomatic infections during the high-incidence period.

The proportionality coefficient *K* reflects the concentration of immature mosquitoes in larval habitats, which is dependent on the climate, environment, hosts, container types, and surveillance methods. Its value can therefore vary from time to time and from place to place. A recent survey carried out in Guangzhou found that the abundance of larva vectors in aquatic habitats fluctuated significantly between urban, suburban, and rural areas, which corresponds to the three different classes of urban, suburban, and exurban [44], so we subdivide the parameter as *K*_1_, *K*_2_, and *K*_3_ accordingly. These parameters are viewed as constants, due to the short period of our study.

#### Daily commuting

To account for remote infections in other locations and to understand the effects of human mobility on dengue transmission, we consider the daily commuting between different locations. A radiation model has recently been developed to simulate human mobility [38], where the commuting is a daily process related to employment, and the radiation model appears to match experimental data very well [38]. In our study, commuters refer to those who live and work (or study) in different districts, and who go out early in the morning and return in the late afternoon. We use the radiation model to estimate commuting frequency in different districts. Let *N*_*i*_ denote the population (number of residents) in district *i*, and *S*_*il*_ be the total population in the circle whose center is the origin *i* and radius is the distance between *i* and the destination *l*, minus the population at *i* and *l*. The number of commuters departing from district *i* to *l* can then be calculated as follows:

$$T_{il}=T_{i}\frac{N_{i}N_{l}}{\left(N_{i}+S_{il}\right)\left(N_{i}+N_{l}+S_{il}\right)}.$$ (3)

Here, *T*_*i*_ is the total number of commuters departing from *i*. It can be formulated as *T*_*i*_ = *N*_*i*_(*N*_*c*_/*N*), where *N*_*c*_ is the total number of commuters and *N* is the total population [38]. Thus, the number of people who physically stay in district *i* at night is ${\overset{ˇ}{N}}_{i}=N_{i}$, while during the daytime the number becomes

$${\hat{N}}_{i}=N_{i}-T_{i}+\sum_{l\neq i}T_{li}.$$

#### Incidence modeling

To determine the potential infectivity from mosquitoes, we use the notion of vectorial capacity (VCAP), which is defined as the average number of infectious mosquito bites per unit time, following the introduction of a single infected host [21, 22]. VCAP can capture the critical components of an insect’s role in pathogen transmission, which is adapted from the basic reproduction number based on the Ross-Macdonald’s model [49]. VCAP has recently been logically generalized to consider mosquito senescence with an age-dependent survival rate and life expectancy [23]. VCAP can therefore be more detailed and precise when evaluating the activities of mosquitoes. Let $V_{i}^{j}$ denote the VCAP contributed by mosquitoes with age *j* in district *i*. Mathematically, it is calculated as [23]:

$$V_{i}^{j}\left(t\right)=m_{i}^{j}\left(t\right)a_{i}^{2}e^{j+k}\prod_{l=j}^{j+k}p\left(l\right),$$ (4)

where *e*^*j*+*k*^ is the expectation of remaining infectious life at age *j* + *k*, and $m_{i}^{j}$ is the ratio of female mosquitos at age *j* to humans, whose values during daytime and nighttime are ${\hat{m}}_{i}^{j}=x_{i}^{j}/{\hat{N}}_{i}$ and ${\overset{ˇ}{m}}_{i}^{j}=x_{i}^{j}/{\overset{ˇ}{N}}_{i}$, respectively.

To further estimate the infection size in each district, we introduce the notion of entomological incubation rate (EIR), which is defined as the number of infectious bites received by a human at each time step [22]. Let ${\hat{y}}_{i}\left(t\right)$ denote the proportion of infected population in the daytime. It is given by ${\hat{y}}_{i}\left(t\right)={\hat{I}}_{i}\left(t\right)/{\hat{N}}_{i}$, where ${\hat{I}}_{i}\left(t\right)$ is the number of infections physically staying in district *i* at time *t*. EIR in the daytime is then calculated through vectorial capacity and temporal prevalence as follows [22]:

$${\hat{E}}_{i}\left(t\right)=\frac{b\left(1-\delta \right){\hat{y}}_{i}\left(t\right)\sum_{j=\sigma }^{h-k}{\hat{V}}_{i}^{j}\left(t\right)}{1+a_{i}be\left(1-\delta \right){\hat{y}}_{i}\left(t\right)},$$ (5)

where $e=\sum_{j=\sigma }^{h-k}ip\left(i\right)$ is the average life span of Aedes mosquitoes. The range of *j* = *σ*, ⋯, *h* − *k* is because only those mosquitoes who can bite and live through EIP can contribute to the infection risk. The EIR at night ${\overset{ˇ}{E}}_{i}\left(t\right)$ can be similarly calculated.

A number of factors determine the selection of some of the parameters. In China, when dengue patients go to hospital for treatment, they are registered and reported according to the regulations. Most of them must be hospitalized or quarantined, and must also take care to avoid being bitten. We therefore assume that the reported cases would not be bitten by mosquitoes again and only the unregistered patients ($1-\delta ){\hat{y}}_{i}$ are involved in the further transmission. Our study time is the high-incidence period of dengue occurrence in Guangzhou, when the government took various measures (e.g., spraying insecticide and cleaning up the environment) to control the dengue outbreak. Information about dengue was also broadcasted widely across the media. Therefore, the treatment rate can be regarded as high and the reported rate as equal to the proportion of symptomatic infections in the total infections.

Based on the definition of EIR, the estimated number of new infections in the daytime is [24]:

$${\hat{L}}_{i}\left(t\right)=c{\hat{E}}_{i}\left(t\right){\hat{N}}_{i}.$$ (6)

Here, we suppose that the total population is susceptible, as those infected and who recovered in Guangzhou only make up a small part of the total population. ${\hat{L}}_{i}\left(t\right)$ counters those infected who physically stay in their district *i* during the daytime. The corresponding value at night ${\overset{ˇ}{L}}_{i}\left(t\right)$ can be derived similarly. The number of new infections in the daytime of those living in district *i* (including those staying away) can therefore be estimated as:

$${\hat{Y}}_{i}\left(t\right)={\hat{L}}_{i}\left(t\right)\left(1-\frac{\sum_{l\neq i}T_{li}}{{\hat{N}}_{i}}\right)+\sum_{l\neq i}{\hat{L}}_{l}\left(t\right)\frac{T_{il}}{{\hat{N}}_{l}}.$$ (7)

Those newly infected in the previous night and who stay in district *i* during the daytime can be calculated as:

$${\overset{ˇ}{Y}}_{i}\left(t\right)={\overset{ˇ}{L}}_{i}\left(t\right)\left(1-\frac{\sum_{l\neq i}T_{il}}{{\overset{ˇ}{N}}_{i}}\right)+\sum_{l\neq i}{\overset{ˇ}{L}}_{l}\left(t\right)\frac{N_{li}}{{\overset{ˇ}{N}}_{l}}.$$ (8)

It should be noted that all new infections ${\hat{L}}_{i}\left(t\right)$ and ${\overset{ˇ}{L}}_{i}\left(t\right)$ are in a latent state at time *t*. Only after the intrinsic incubation period (IIP) (3–7days [46, 50]), they will become patients (with or without symptoms) and *δ* of them will seek treatment.

To activate the formula (5), temporal prevalences $\hat{I}$ and $\overset{ˇ}{I}$ is estimated as follows:

$$\begin{array}{c} {\hat{I}}_{i}\left(t\right)=\sum_{l=1}^{\tau }P\left(l\right)\left(\sum_{j=1}^{d}P_{L}\left(j\right)\left({\hat{L}}_{i}\left(t-j-l\right)+{\overset{ˇ}{Y}}_{i}\left(t-j-l\right)\right)\right),\\ {\overset{ˇ}{I}}_{i}\left(t\right)=\sum_{l=1}^{\tau }P\left(l\right)\left(\sum_{j=1}^{d}P_{L}\left(j\right)\left({\overset{ˇ}{L}}_{i}\left(t-j-l\right)+{\hat{Y}}_{i}\left(t-j-l\right)\right)\right), \end{array}$$ (9)

where *P*_*L*_(*j*) is the probability that the individuals become infected after being bitten *j* days before, and $P\left(l\right)=1-\sum_{i=1}^{l}P_{I}\left(i\right)$ is the probability that those individuals who become infected *l* days before remain infected. *τ* and *d* denote the longest IIP and the maximum infection period, respectively.

Based on the report cards of infectious diseases in China, patients are registered as incidence cases in their own residential districts. This may introduce a spatial mismatch between the surveillance data and real infections. Hence, to evaluate the real transmission level, any remote infections should be projected into their residential districts. Based on the radiation model (3) and the transmission equations presented above, the number of real incidences among people living in district *i* can be calculated as follows:

$$\rho_{i}\left(t\right)=\sum_{l=1}^{d}P_{L}\left(l\right)\left({\hat{Y}}_{i}\left(t-l\right)+\overset{ˇ}{L}\left(t-l\right)\right).$$ (10)

So far, the standard transmission process has been formulated by Eqs (2–10). To implement this model in a specific region, knowledge of the BI values, the initial number of infections, and suitable parameters is needed. The initial infections can be estimated by dividing the number of the reported cases by a reported rate, and undetermined parameters can be fitted by machine learning methods.

#### Markov chain Monte Carlo method

We adopt a Markov chain Monte Carlo (MCMC) method to estimate the model parameters. The model parameters *K* and *a* are estimated by fitting our model with surveillance data. The relationship between the sizes of reported cases and of modeling cases can be written in matrix notation as Γ = *δρ*+*ε*, where Γ = (Γ_1_, Γ_2_, ⋯, Γ_*H*_) is a *H* × *T* matrix, representing the surveillance data, and *ρ* = (*ρ*_1_, *ρ*_2_, ⋯, *ρ*_*H*_) is a *H* × *T* matrix, representing the modeling incidences. The *H* × *T* matrix *ε* follows a matrix normal distribution, i.e., *ε* ∼ *N*(0, *I*_*T*_, Σ). To account for any misalignment of the report date, each element in Γ equals the average of reported cases over two successive days.

The likelihood can be calculated as:

$$P\left(\Gamma |K,a\right)={\left(2\pi \right)}^{-HT/2}{\left|\Sigma \right|}^{-T/2}\exp\left(tr\left(\frac{{\left(\Gamma -\delta \rho \right)}^{\prime }\left(\Gamma -\delta \rho \right)}{2\Sigma }\right)\right),$$ (11)

where in the evolution dynamic process *K* = (*K*_1_, *K*_2_, *K*_3_), *a* = (*a*_1_, *a*_2_, ⋯, *a*_*H*_). Accordingly, the joint posterior distributions of *K* and *a* are given by

$$P\left(K,a|\Gamma \right)\propto \prod_{t=1}^{T}P\left(\Gamma_{t}|\rho_{t},K,a\right)\prod_{i=1}^{3}P\left(K_{i}\right)\prod_{i=1}^{H}P\left(a_{i}\right).$$ (12)

The procedure of the MCMC method is carried out as follows [39]: First, we initialize all of the independent model parameters *K* and *a*, each of which follows a normal distribution. We then generate the value of the modeling cases based on new parameters for calculating the posteriori likelihood *P*(K*, a*|Γ) according to Eq (11). For each iteration, new values of Γ are generated from an adaptive proposal distribution *P*(K*, a*|*K*, *a*). New values of *K* and *a* can then be calculated. All new values K*, A* and Γ* will be accepted with probability

$$\min\left(1,\frac{P\left(K^{*},a^{*}|\Gamma \right)q\left(K,a|K^{*},a^{*}\right)}{P\left(K,a|\Gamma \right)q\left(K^{*},a^{*}|K,a\right)}\right),$$

where *q*(K*, a*|*K*, *a*) is the proposed density. After a number of iterations, we can then analyze the statistics of the model parameters and estimate their values.

## Results

### The heterogeneity of vector behaviors

According to the proposed model and the MCMC algorithm, the underlying model parameters are estimated, and the values are presented in Table 2. Based on the scale factors between BI and mosquito density (i.e., *K*_1_, *K*_2_ and *K*_3_), it is observed that the aquatic habitats contain the highest concentration of larval mosquitoes in urban areas and the lowest concentration in exurban areas, but the difference between the exurban and suburban areas is not significant. This finding is consistent with the results in [44], where the authors have found that in urban areas of Guangzhou, the larvae and pupae of Aedes albopictus are more abundant in container habitats. The possible reason for the disparity of *K* is that the temperature, food sources, and types of aquatic habitats and containers vary between the urban, suburban, and rural areas [44].

**Table 2:** Posterior means with posterior standard deviation (SD) for model parameters.

Parameters	Mean (SD)	SD	Parameters	Mean	SD	Parameters	Mean	SD
*K*_1_^1^	12347	53	*a*_3_	0.332	0.007	*a*_8_	0.246	0.021
*K*_2_	7976	68	*a*_4_	0.485	0.012	*a*_9_	0.282	0.014
*K*_3_	6521	37	*a*_5_	0.428	0.011	*a*_10_	0.237	0.013
*a*_1_^2^	0.391	0.006	*a*_6_	0.286	0.005	*a*_11_	0.318	0.012
*a*_2_	0.361	0.022	*a*_7_	0.271	0.01	*a*_12_	0.267	0.011

^1^Parameters *K*_1_, *K*_2_, *K*_3_ are the scale factors between the Breteau index and vector density in the urban, suburban and exurb areas.

^2^Parameter *a*_*i*_ (*i* = 1, 2, ⋯, 12) is the Aedes mosquitoes biting rate of humans in district *i* in Guangzhou within 12 hours, where districts 1 to 12 correspond to Yuexiu, Haizhu, Liwan, Tianhe, Baiyun, Panyu, Huangpu, Luogang, Huadu, Nansha, Zengcheng, and Conghua, respectively.

The estimated number of bites per person per 12 hours by each female Aedes mosquito is shown in Table 2, presented as *a*_*i*_ in district *i*. This rate is equal to the product of the human blood index (i.e., the proportion of blood meals of mosquitoes taken from humans) and the mosquito feeding frequency, which is possibly associated with the status of demography, temperature, geography and environment [42]. In Guangzhou, the terrain slopes downward from the north to the south. The temperature usually falls 1–2 degree from the south to the north, but the urban heat island effect results in over 1.7 higher degrees in the center areas in 2014. Low latitude and high temperature can cause frequent mosquito feeding. Specifically, our results indicate that urban areas possess high values of human blood index or mosquito feeding frequency. This is perhaps due to the dense population and the urban heat island effect. Tianhe is the most prosperous district (with the highest GDP) and one of the densely populated areas. Mosquitoes there prefer to bite humans frequently. In the suburban and exurban areas, however, probably due to various blood sources (e.g., chicken, dogs, and cattle), cool temperature, high altitude, and the sparse population, the biting rate on humans is relatively low.

### The infection risk

**Fig 3:**
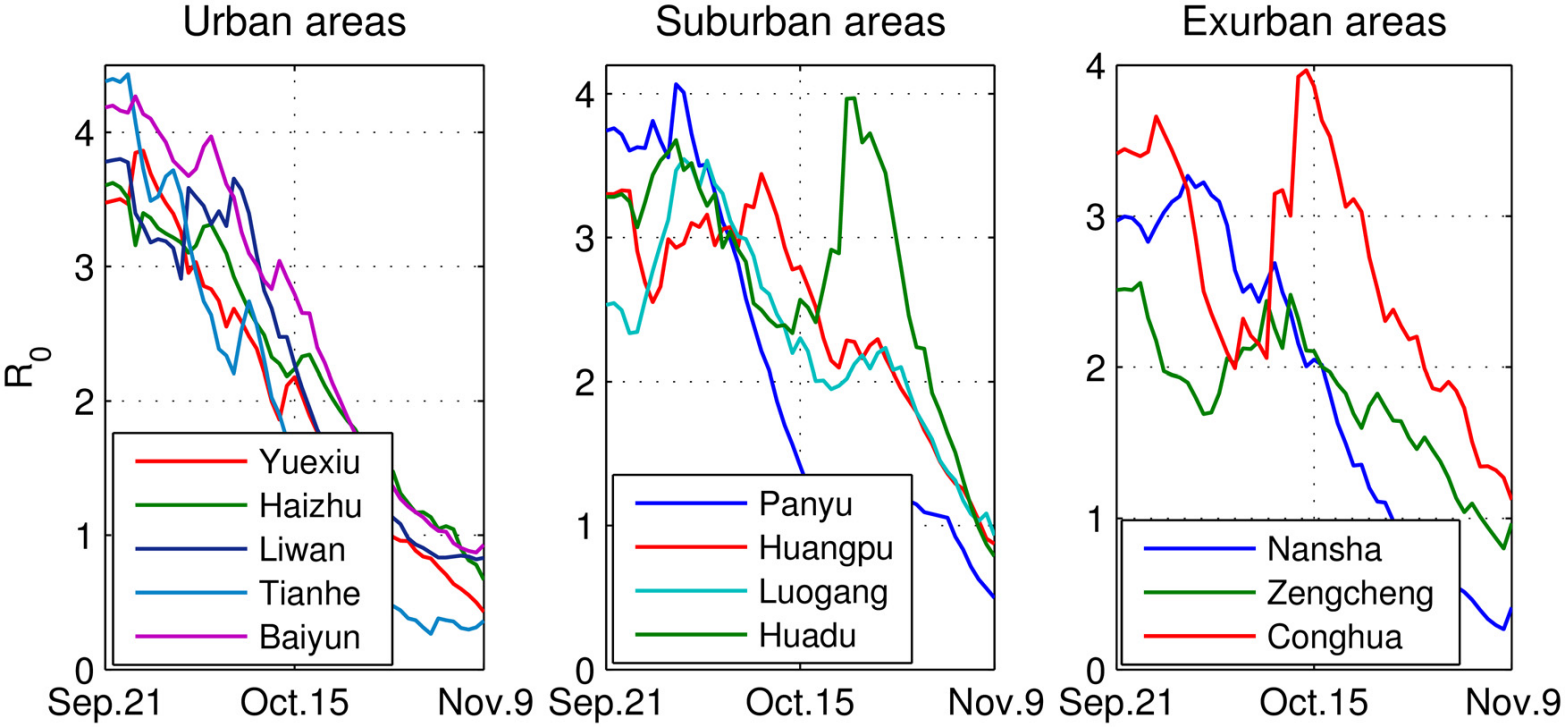
The basic reproduction number *R*_0_ in each district of Guangzhou. The solid lines are the longitudinal *R*_0_ from September 21 to November 9, 2014. The numbers in the legends are the average *R*_0_ during this period in the corresponding districts. Here, a portion of Baiyun is in the suburban area. The basic reproduction number reflects the transmission potential of an epidemic disease.

The infection risk is usually evaluated by the basic reproduction number *R*_0_, defined as the expected number of secondary infections averagely generated by one case in a completely susceptible population [9, 19, 22]. *R*_0_ is widely used as an invasion threshold: if *R*_0_ is less than one, then the disease will become extinct; otherwise, there will exist an endemic state. In epidemiology, *R*_0_ reflects the biology of the transmission dynamics and quantifies the transmission potential of an epidemic. For vector-borne diseases, the basic reproduction number was first derived by Macdonald (1957) and Ross (1911) [9], based on which, we present the following formula:

$$R_{0}=4bc\vartheta \sum_{j=\sigma }^{h-k}V^{j},$$ (13)

where *ϑ* = ∑_*l*_ *lP*_*I*_(*l*) = 5 days [48] is the average duration of human infection. Eq (13) is an evolutionary form of the basic reproduction number with time-varied VCAP. Averaging over the vector densities through the study period and inserting it into Eq (13), we obtain the mean value of the basic reproduction number *R*_0_. The evolutionary and average values of *R*_0_ in each district of Guangzhou are presented in Fig 3. It can be observed that *R*_0_ decreases from the largest value, 4.4 (in Tianhe), in late September to less than 1 in early November, and the average value is between 1.81–2.59. We find that *R*_0_ is much higher but decreases more quickly in urban areas, which implies that the infection capacity is at first greater in urban areas, and the following intervention measures are effective there. Two peaks of *R*_0_ are observed in Huadu and Conghua, due to the increase in mosquito numbers. It should be noted that a larger *R*_0_ does not determine a higher incidence, and a rapid decrease of *R*_0_ does not means a rapid reduction in incidences, as the incidence rate is also dependent on the infection sources.

### The spatio-temporal patterns of dengue dynamics

**Fig 4:**
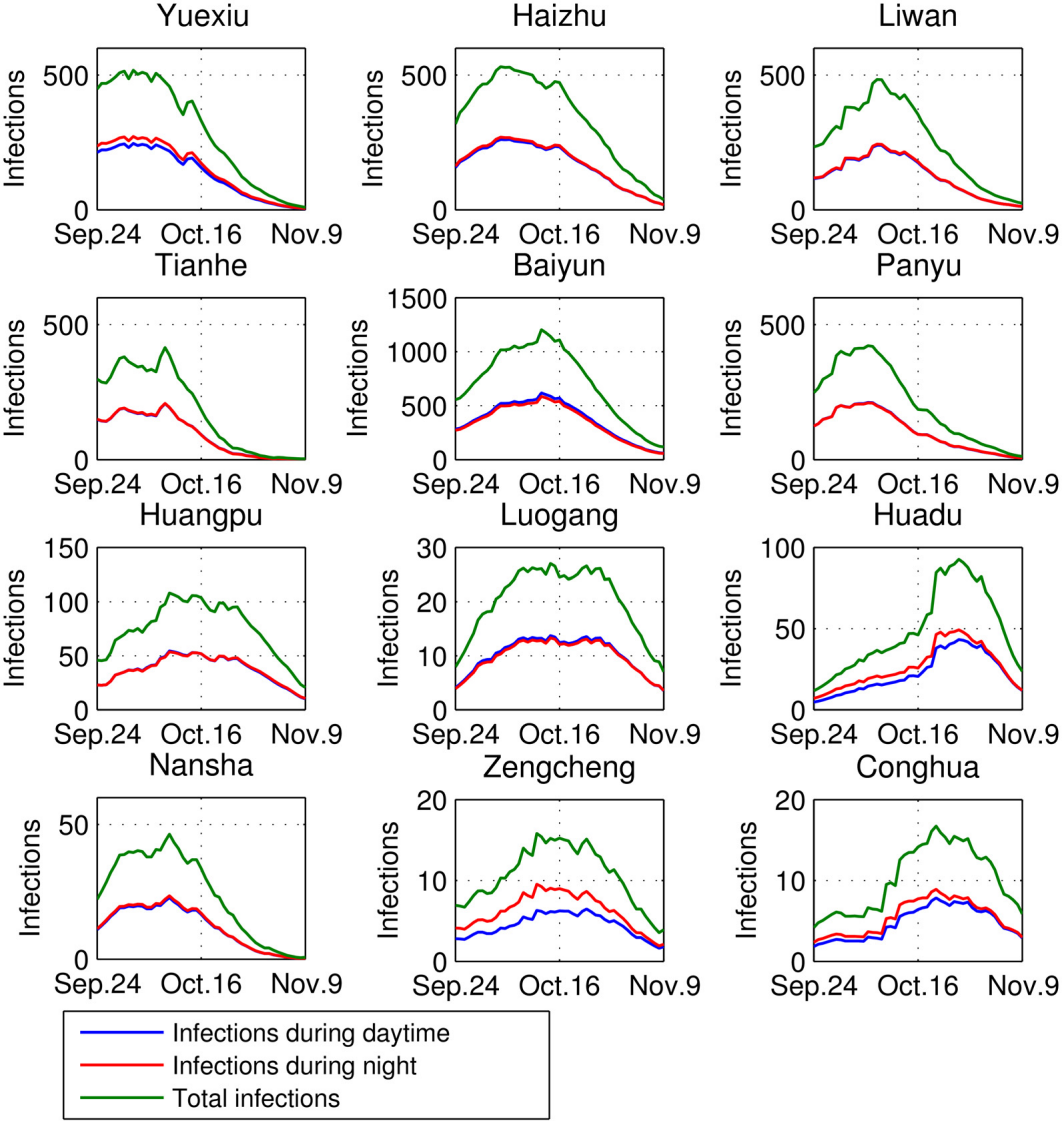
Daily infections with the difference between daytime and nighttime in 12 districts, Guangzhou. The time span is from September 24 to November 9, 2014. The time corresponds to the moments when people are bitten and get infected, so these patients are in a latent state. Some patients are infected in other districts.

**Fig 5:**
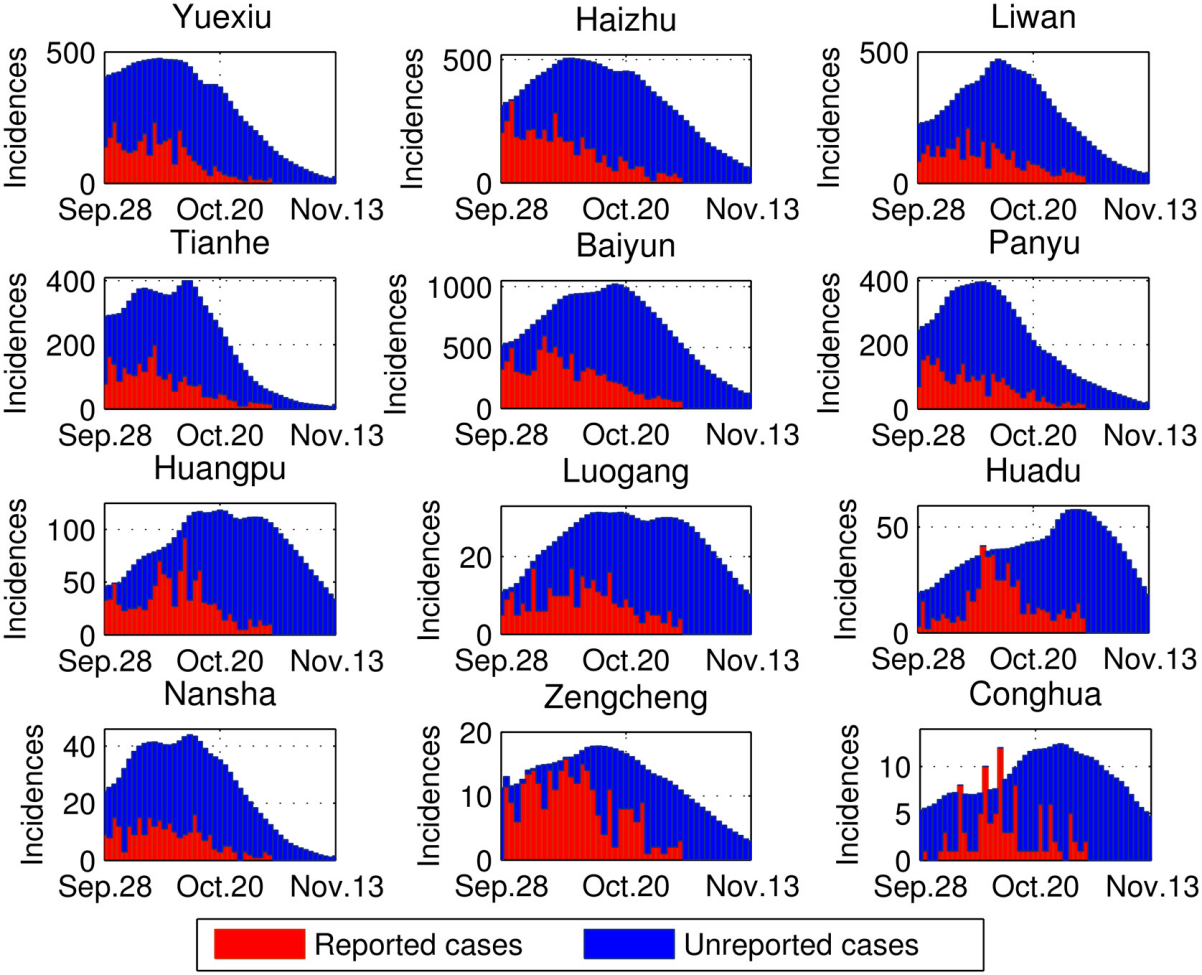
Comparison of estimated cases and reported cases in 12 districts, Guangzhou. The time span is from September 28 to November 13, 2014. The daily reported cases are available and demonstrated between September 28 and October 31 only.

**Fig 6:**
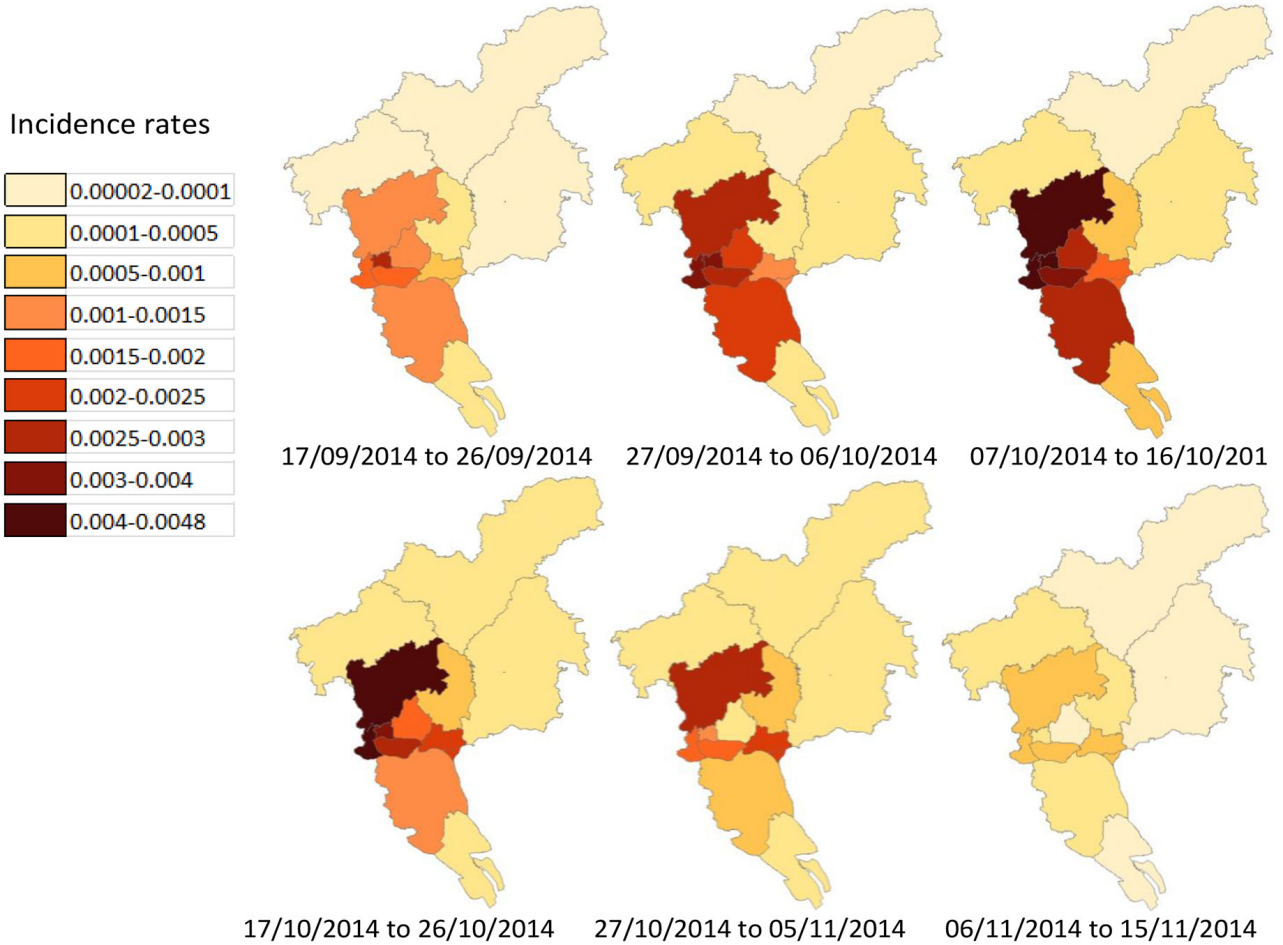
The estimated spatio-temporal patterns of incidence rates. The incidence rate in a particular district is computed as the proportion between the infection size and the number of people who physically stay in the region.

Applying the estimated parameters to the proposed model, we obtain the following estimation: (1) The longitudinal number of infections in each district with the infection difference between daytime and night is as shown in Fig 4, in which the time corresponds to when people are bitten and get infected; (2) The longitudinal numbers of the incidences are as shown in Fig 5, in which the reported cases are demonstrated as a part of them; (3) The levels of the remote and local infections are as summarized in Table 3, in which the living areas and bit locations of the patients are estimated; (4) The spatio-temporal incidence rates are as shown in Fig 6. From the above Figures and Table, we observe heterogeneous and interesting patterns in dengue transmission in these districts, which are specified from the following aspects.

**Table 3:** The number of dengue cases based on remote and local infection among 12 districts in Guangzhou, with the infections occurring from September 24 to November 9, 2014.

	Yuexiu	Haizhu	Liwan	Tianhe	Baiyun	Panyu	Huangpu	Luogang	Huadu	Nansha	Zengcheng	Conghua	Total
Yuexiu	14560	172	189	96	119	21	20	4	5	1	0	0	15187
Haizhu	401	16608	319	150	128	56	31	5	3	1	1	0	17703
Liwan	241	104	12471	45	61	12	10	1	1	0	0	0	12946
Tianhe	326	346	121	9612	247	40	38	5	2	1	0	0	10738
Baiyun	459	323	264	346	32814	52	60	20	17	2	1	1	34359
Panyu	150	439	114	87	71	10423	46	3	2	4	0	0	11294
Huangpu	15	23	7	40	11	4	3751	1	0	0	0	0	3852
Luogang	10	21	9	29	27	3	32	1026	0	0	0	0	1157
Huadu	37	36	29	35	340	5	7	3	2419	0	0	0	2911
Nansha	6	21	5	3	6	55	5	0	0	1261	0	0	1362
Zengcheng	22	36	19	61	79	13	60	30	2	1	680	0	1003
Conghua	8	11	4	9	39	2	4	4	1	0	1	513	596
Total	16190	18140	13551	10513	33942	10686	4064	1102	2452	1271	683	514	113108

The element (*i*, *j*) in this table corresponds to those people who live in district *i* but are infected in district *j*.

First, the number of infections is estimated at about 113,108 cases from September 24 to November 9, 2014, some of which are recorded in the surveillance system. Most of the unreported cases are inapparent. We can classify the 12 districts into 3 classes.

- The areas with the highest incidence rates are around the urban center (i.e., Yuexiu, Haizhu, Liwan, Tianhe, and Baiyun), where about 92,336 people (81.6% of the total cases) are infected. The obvious hotspots can be observed in the areas around Liwan, Yuexiu, and Baiyun. The primarily reasons for the high incidences in the urban center could be: (1) high concentrations of people (about 57% of residents and 17% of the city’s area), (2) a highly fluid population (with an estimated 81.7% daily mobility rate in these 5 districts), (3) abundant initial infections, and (4) high vector density and frequent human biting.
- The areas with the second highest incidence rates make up one section of the suburban area (i.e., Panyu and Huangpu). These two adjacent districts are close to the incidence hotspot, and many residents work or study in the central areas.
- The five remaining districts demonstrate relatively small infection levels, (6,022 cases, 5.3% of the total cases). These districts are located on the border of Guangzhou, where the human mobility rate is quite low and the infection source is small. In this case, high vector indices do not lead to high incidences.

Second, the patterns of infection differences between daytime and nighttime are shown in Fig 4. A slightly higher infection rate can be observed at night for residents in the urban center, which may be due to people returning to their living districts in late evening, and the infections risk is relatively high in urban areas. However, in most suburban and exurban areas, the lower infection risk creates a lower probability of getting infected at night. If the number of people leaving a certain district each day is larger than the number moving in (i.e., in Baiyun, Panyun, Huadu, Nansha, Zengcheng, and Conghua), then the incidence rate in the daytime could be higher due to remote infections.

Third, typical temporal patterns of infection are summarized as follows:

- All 12 districts experience a fluctuation in terms of dengue incidences, but the peak time varies, with the first October 11 (Yuexiu) and the last October 30 (Huadu). These phenomena are due to the diverse levels of infection sources (i.e., infected people and vectors) and the heterogeneous activities of Aedes mosquitoes.
- Population density and mobility play significant roles in dengue diffusion. For example, based on surveillance data, Yuexiu, Baiyun, and Nansha are the first to report 100 cases, but the transmission patterns are different. Low mobility yields a small incidence level in Nansha. But Baiyun as the area with the largest numbers of residents and commuters, it has the most incidences and a comparatively late peak.
- The continuing rapid decline of BI values results in a relatively rapid decline in the incidence rate, in Yuexiu, Panyu, and Nansha, and particularly in Tianhe. This result also indicates that intervention in these districts was timely and effective.
- Double peaks are observed in some districts, such as Tianhe and Nansha, due to the fluctuation of vector densities and occurrence peak incidences in other districts. The latest peak incidences occur in Huadu and Conghua, as they are the last districts involved in the transmission, and the BI values are still high in late October.

### The diffusion route

Based on the surveillance data, the weight of human mobility, the quantity of remote incidences, and the arriving time of incidence peak, particularly the spatio-temporal incidence rates, we are able to identify the primary route of dengue diffusion in Guangzhou.

- The first step refers to the spread through central areas: Local dengue infection initially takes place in Yuexiu, the biggest remote infection source, which then triggers a rapid spreading of the dengue virus through Liwan, Baiyun, and then invades Haizhu. Tianhe and Panyu are at the same time involved in the transmission process, due to the interchange of infection sources.
- The second step refers to the spreading process through the periphery of the central areas. Remote infections from the central areas gradually cause widespread transmission in suburban and exurban areas. Nansha, Zengcheng, Huangpu, and Luogang experience the infection process at a similar rate, while Huadu and Conghua suffer infections slightly later. During transmission, Nansha, Zengcheng, Huadu, and Conghua contribute few infections to other areas.

It should be noted that the effects of human mobility are not just reflected in the remote infections, and by introducing infectivity to local Aedes mosquitoes, human mobility can lead to large number of autochthonous dengue cases.

## Discussion

In this study, we have developed an inference technique to identify dengue transmission patterns and applied it to the 2014 dengue outbreak in Guangzhou, China. From this approach, we can improve our understanding of the dengue burden, infection risk, and the transmission dynamics in Guangzhou. Our results can help policy makers formulate effective measures to control and prevent dengue transmission.

Our model is based on the Ross-Macdonald theory, which can be viewed as an epidemiological compartment model with an SEI infection process. The model closely combines four key sub-models necessary for describing the integrated dynamics of the system, namely, those representing mosquito population dynamics, human movement, virus transmission, and parameters estimation. First, we present a novel method to estimate the quantities of adult female mosquitoes from the BI data and environmental information. This approach differs from other studies that directly use vector indices and involved factors (e.g., climate, sociodemographic indicators, and land-cover types) to estimate the potential dengue risk from a statistical perspective [14, 16, 26]. Second, based on the available transportation data, we use a standard radiation model to approximate the human mobility pattern [38]. As an inevitable component in dengue spatial transmission, human mobility can also be tracked by many other methods, such as GPS data [27], agent-based models, [25], and metapopulation models [10]. Next, we integrate well-recognized formulas (e.g., the vectorial capacity [21–23] and the entomological incubation rate [22, 24]) to elucidate the transmission process. We take into account the spatial heterogeneity of vector-host interactions, and the corresponding biting rates are estimated by MCMC methods. Our model further explore the real dynamics of disease transmission behind the observed incidences. The framework can also be applied to the space-time analysis of other vector-borne diseases.

Based on our empirical study in Guangzhou, we find that the spatio-temporal distribution of incidences is extremely heterogeneous, with 81.6% infections occurring in urban centers with different shapes of peak existing in mid-October. By considering the underlying dynamics, we observe temporal and spatial disagreement between infection cases and reported cases. The rank of disease burden in 12 districts is also inconsistent with the surveillance results.

Further, We find that, in Guangzhou, the basic reproduction number *R*_0_ as an indicator of the infection risk decreases from the peak (3.45) on September 22 to the trough (0.73) on November 9, 2014, with a mean value of 2.24. This estimated *R*_0_ can be applied to quantify the infectivity in 12 districts and measure the effectiveness of the control strategies. From September, the Guangzhou government began to adopt various measures to control dengue transmission, such as disseminating knowledge about dengue through different media, asking every family to clean and clear their water containers, and organizing a sweep each Friday and regular spring-cleaning throughout the city. Consequently, we find that *R*_0_ begins to decrease from late September in most areas, particularly urban regions. However, due to a large number of infectious sources, the incidences decreased in about mid-October. This indicates that to control dengue transmission, intervention measures must be taken in a timely fashion.

Due to the availability and validity of current surveillance data, the proposed models have certain limitations, which are worthy of further improvement and discussion: (1) People in Guangzhou are assumed to be without immunity against dengue; (2) The biological parameters are extracted from the literature (see Table 1), which may show geographical disparities; (3) The reported rate is used as the the proportion of symptomatic infections from [4]. Further experiments and survey are necessary to validate these parameters.

## References

[1] World Health Organization. Dengue and severe dengue; 2015 Available http://www.who.int/mediacentre/factsheets/fs117/en/.

[2] World Health Organization. Dengue: guidelines for diagnosis, treatment, prevention and control. WHO; 2009. 23762963

[3] World Health Organization. Global strategy for dengue prevention and control 2012–2020. WHO; 2012.

[4] Bhatt S, Gething PW, Brady OJ, Messina JP, Farlow AW, Moyes CL, et al The global distribution and burden of dengue. Nature. 2013 4;496(7446):504–507. 10.1038/nature12060 23563266 PMC3651993

[5] Lai S, Huang Z, Zhou H, Anders KL, Perkins TA, Yin W, et al The changing epidemiology of dengue in China, 1990–2014: a descriptive analysis of 25 years of nationwide surveillance data. BMC Medicine. 2015 4;13(1):100 10.1186/s12916-015-0336-1 25925417 PMC4431043

[6] Montoya M, Gresh L, Mercado JC, Williams KL, Vargas MJ, Gutierrez G, et al Symptomatic versus inapparent outcome in repeat dengue virus infections is influenced by the time interval between infections and study year. PLoS Negl Trop Dis. 2013 8;7(8):e2357 10.1371/journal.pntd.0002357 23951377 PMC3738476

[7] Chan TC, Hu TH, Hwang JS. Daily forecast of dengue fever incidents for urban villages in a city. Int J Health Geogr. 2015 1;14:9 10.1186/1476-072X-14-9 25636965 PMC4351941

[8] Wang T, Wang M, Shu B, Chen XQ, Luo L, Wang J, et al Evaluation of inapparent dengue infections during an outbreak in Southern China. PLoS Negl Trop Dis. 2015 3;9(3):e0003677 10.1371/journal.pntd.0003677 25826297 PMC4380470

[9] Smith DL, Battle KE, Hay SI, Barker CM, Scott TW, McKenzie FE. Ross, Macdonald, and a theory for the dynamics and control of mosquito-transmitted pathogens. PLoS pathog. 2012 4;8(4):e1002588 10.1371/journal.ppat.1002588 22496640 PMC3320609

[10] Adams B, Kapan D, Galvani AP. Man bites mosquito: understanding the contribution of human movement to vector-borne disease dynamics. PLoS ONE. 2009 8;4(8):e6763 10.1371/journal.pone.0006763 19707544 PMC2727792

[11] O’Neill PD, Roberts GO. Bayesian inference for partially observed stochastic epidemics. J R Stat Soc Ser A Stat Soc. 1999 1;162(1):121–129. 10.1111/1467-985X.00125

[12] Ster IC, Singh BK, Ferguson NM. Epidemiological inference for partially observed epidemics: the example of the 2001 foot and mouth epidemic in Great Britain. Epidemics. 2009 3;1(1):21–34. 10.1016/j.epidem.2008.09.001 21352749

[13] Cooper B, Lipsitch M. The analysis of hospital infection data using hidden Markov models. Biostatistics. 2004 4;5(2):223–237. 10.1093/biostatistics/5.2.223 15054027

[14] Bowman LR, Runge-Ranzinger S, McCall P. Assessing the relationship between vector indices and dengue transmission: a systematic review of the evidence. PLoS Negl Trop Dis. 2014 5;8(5):e2848 10.1371/journal.pntd.0002848 24810901 PMC4014441

[15] Sivagnaname N, Gunasekaran K. Need for an efficient adult trap for the surveillance of dengue vectors. Indian J Med Res. 2012 11;136(5):739 23287120 PMC3573594

[16] Vargas WP, Kawa H, Sabroza PC, Soares VB, Honório NA, de Almeida AS. Association among house infestation index, dengue incidence, and sociodemographic indicators: surveillance using geographic information system. BMC Public Health. 2015 8;15(1):746 10.1186/s12889-015-2097-3 26243266 PMC4526415

[17] Tun-Lin W, Kay B, Barnes A, Forsyth S. Critical examination of Aedes aegypti indices: correlations with abundance. Am J Trop Med Hyg. 1996 5;54(5):543–547. 8644913 10.4269/ajtmh.1996.54.543

[18] Focks DA. A review of entomological sampling methods and indicators for dengue vectors. Geneva: WHO 2003.

[19] Chowell G, Diaz-Dueñas P, Miller J, Alcazar-Velazco A, Hyman J, Fenimore P, et al Estimation of the reproduction number of dengue fever from spatial epidemic data. Math Biosci. 2007 8;208(2):571–589. 10.1016/j.mbs.2006.11.011 17303188

[20] Khan A, Hassan M, Imran M. Estimating the basic reproduction number for single-strain dengue fever epidemics. Infect Dis Poverty. 2014 4;3(1):12 10.1186/2049-9957-3-12 24708869 PMC4021574

[21] Lambrechts L, Paaijmans KP, Fansiri T, Carrington LB, Kramer LD, Thomas MB, et al Impact of daily temperature fluctuations on dengue virus transmission by Aedes aegypti. Proc Natl Acad Sci USA. 2011 5;108(18):7460–7465. 10.1073/pnas.1101377108 21502510 PMC3088608

[22] Smith DL, McKenzie FE. Statics and dynamics of malaria infection in Anopheles mosquitoes. Malaria J. 2004 6;3(1):13 10.1186/1475-2875-3-13 PMC449722 15180900

[23] Styer LM, Carey JR, Wang JL, Scott TW. Mosquitoes do senesce: departure from the paradigm of constant mortality. Am J Trop Med Hyg. 2007 1;76(1):111–117. 17255238 PMC2408870

[24] Shi B, Liu J, Zhou XN, Yang GJ. Inferring plasmodium vivax transmission networks from tempo-spatial surveillance data. PLoS Negl Trop Dis. 2014 2;8(2):e2682 10.1371/journal.pntd.0002682 24516684 PMC3916251

[25] Mammen MP Jr, Pimgate C, Koenraadt CJ, Rothman AL, Aldstadt J, Nisalak A, et al Spatial and temporal clustering of dengue virus transmission in Thai villages. PLoS Med. 2008 11;5(11):e205 10.1371/journal.pmed.0050205 18986209 PMC2577695

[26] Sarfraz MS, Tripathi NK, Tipdecho T, Thongbu T, Kerdthong P, Souris M. Analyzing the spatio-temporal relationship between dengue vector larval density and land-use using factor analysis and spatial ring mapping. BMC Public Health. 2012 10;12(1):853 10.1186/1471-2458-12-853 23043443 PMC3598814

[27] Vazquez-Prokopec GM, Stoddard ST, Paz-Soldan V, Morrison AC, Elder JP, Kochel TJ, et al Usefulness of commercially available GPS data-loggers for tracking human movement and exposure to dengue virus. Int J Health Geogr. 2009 11;8:68 10.1186/1476-072X-8-68 19948034 PMC2792221

[28] Wen TH, Lin NH, Lin CH, King CC, Su MD. Spatial mapping of temporal risk characteristics to improve environmental health risk identification: a case study of a dengue epidemic in Taiwan. Sci Tot Environ. 2006 8;367(2):631–640. 10.1016/j.scitotenv.2006.02.009 16584757

[29] Sang S, Yin W, Bi P, Zhang H, Wang C, Liu X, et al Predicting local dengue transmission in Guangzhou, China, through the influence of imported cases, mosquito density and climate variability. PLoS ONE. 2014 7;9(7):e102755 10.1371/journal.pone.0102755 25019967 PMC4097061

[30] Nishiura H. Mathematical and statistical analyses of the spread of dengue. Dengue Bull. 2006 12;30:51–67.

[31] Racloz V, Ramsey R, Tong S, Hu W. Surveillance of dengue fever virus: a review of epidemiological models and early warning systems. PLoS Negl Trop Dis. 2012 5;6(5):e1648 10.1371/journal.pntd.0001648 22629476 PMC3358322

[32] Wu PC, Lay JG, Guo HR, Lin CY, Lung SC, Su HJ. Higher temperature and urbanization affect the spatial patterns of dengue fever transmission in subtropical Taiwan. Sci Tot Environ. 2009 3;407(7):2224–2233. 10.1016/j.scitotenv.2008.11.034 19157509

[33] Fan J, Lin H, Wang C, Bai L, Yang S, Chu C, et al Identifying the high-risk areas and associated meteorological factors of dengue transmission in Guangdong Province, China from 2005 to 2011. Epidemiol Infect. 2014 3;142(03):634–643. 10.1017/S0950268813001519 23823182 PMC9161228

[34] Toan DTT, Hu W, Thai PQ, Hoat LN, Wright P, Martens P. Hot spot detection and spatio-temporal dispersion of dengue fever in Hanoi, Vietnam. Global Health Action. 2013 1;6:18632 10.3402/gha.v6i0.18632 23364076 PMC3556563

[35] Li Z, Yin W, Clements A, Williams G, Lai S, Zhou H, et al Spatiotemporal analysis of indigenous and imported dengue fever cases in Guangdong province, China. BMC Infect Dis. 2012 6;12(1):132 10.1186/1471-2334-12-132 22691405 PMC3412724

[36] Reiner RC, Stoddard ST, Scott TW. Socially structured human movement shapes dengue transmission despite the diffusive effect of mosquito dispersal. Epidemics. 2014 3;6:30–36. 10.1016/j.epidem.2013.12.003 24593919 PMC3971836

[37] Reiner RC, Perkins TA, Barker CM, Niu T, Chaves LF, Ellis AM, et al A systematic review of mathematical models of mosquito-borne pathogen transmission: 1970–2010. J R Soc Interface. 2013 2;10(81):20120921 10.1098/rsif.2012.0921 23407571 PMC3627099

[38] Simini F, González MC, Maritan A, Barabási AL. A universal model for mobility and migration patterns. Nature. 2012 2;484(7392):96–100. 10.1038/nature10856 22367540

[39] Shi B, Tan Q, Zhou XN, Liu J. Mining geographic variations of Plasmodium vivax for active surveillance: a case study in China. Malaria J. 2015 5;14(1):216 10.1186/s12936-015-0719-y PMC4450990 26013665

[40] Morin CW, Comrie AC, Ernst K. Climate and dengue transmission: evidence and implications. Env Health Persp. 2013 11;121(11–12):1264–1272. 10.1289/ehp.1306556 PMC3855512 24058050

[41] Chen CD. Biting behavior of Malaysian mosquitoes, Aedes albopictus Skuse, Armigeres kesseli Ramalingam, Culex quinquefasciatus Say, and Culex vishnui Theobald obtained from urban residential areas in Kuala Lumpur. Asian Biomed. 2014 6;8(3):315–321.

[42] Almeida APG, Baptista SS, Sousa CA, Novo MTL, Ramos HC, Panella NA, et al Bioecology and vectorial capacity of Aedes albopictus (diptera: culicidae) in Macao, China, in relation to dengue virus transmission. J Med Entomol. 2005 5;42(3):419–428. 15962796 10.1093/jmedent/42.3.419

[43] Sumodan P. Observations on nocturnal endophagy in Aedes (stegomyia) albopictus (skuse), 1894 from Kerala, India. J Entomol Zool Stud. 2014 9;2(5):45–47.

[44] Li Y, Kamara F, Zhou G, Puthiyakunnon S, Li C, Liu Y, et al Urbanization increases Aedes albopictus larval habitats and accelerates mosquito development and survivorship. PLoS Negl Trop Dis. 2014 11;8(11):e3301 10.1371/journal.pntd.0003301 25393814 PMC4230920

[45] Tjaden NB, Thomas SM, Fischer D, Beierkuhnlein C. Extrinsic incubation period of dengue: knowledge, backlog, and applications of temperature dependence. PLoS Negl Trop Dis. 2013 6;7(6):e2207 10.1371/journal.pntd.0002207 23826399 PMC3694834

[46] Nishiura H, Halstead SB. Natural history of dengue virus (DENV)-1 and DENV-4 infections: aeanalysis of classic studies. J Infect Dis. 2007 4;195(7):1007–1013. 10.1086/511825 17330791

[47] Schmidt WP, Suzuki M, Thiem VD, White RG, Tsuzuki A, Yoshida LM, et al Population density, water supply, and the risk of dengue fever in Vietnam: cohort study and spatial analysis. PLoS Med. 2011 8;8(8):e1001082 10.1371/journal.pmed.1001082 21918642 PMC3168879

[48] Gubler DJ. Dengue and dengue hemorrhagic fever. Clin Microbiol Rev. 1998 7;11(3):480–496. 9665979 10.1128/cmr.11.3.480 PMC88892

[49] Massad E, Coutinho FAB. Vectorial capacity, basic reproduction number, force of infection and all that: formal notation to complete and adjust their classical concepts and equations. Mem Inst Oswaldo Cruz. 2012 6;107(4):564–567. 10.1590/S0074-02762012000400022 22666873

[50] Chan M, Johansson MA. The incubation periods of dengue viruses. PLoS ONE. 2012 11;7(11):e50972 10.1371/journal.pone.0050972 23226436 PMC3511440

